# Structure of protease-cleaved *Escherichia coli* α-2-macroglobulin reveals a putative mechanism of conformational activation for protease entrapment

**DOI:** 10.1107/S1399004715008548

**Published:** 2015-06-30

**Authors:** Cameron D. Fyfe, Rhys Grinter, Inokentijs Josts, Khedidja Mosbahi, Aleksander W. Roszak, Richard J. Cogdell, Daniel M. Wall, Richard J. S. Burchmore, Olwyn Byron, Daniel Walker

**Affiliations:** a Institute of Infection, Immunity and Inflammation, College of Medical, Veterinary and Life Sciences, University of Glasgow, Glasgow G12 8QQ, Scotland; b WestCHEM, School of Chemistry, College of Science and Engineering, University of Glasgow, Glasgow G12 8QQ, Scotland; c Institute of Molecular Cell and Systems Biology, College of Medical, Veterinary and Life Sciences, University of Glasgow, Glasgow G12 8QQ, Scotland; d School of Life Sciences, College of Medical, Veterinary and Life Sciences, University of Glasgow, Glasgow G12 8QQ, Scotland

**Keywords:** α-2-macroglobulin, protease inhibitor, conformational change, intrinsic disorder

## Abstract

Bacterial α-2-macroglobulins have been suggested to function in defence as broad-spectrum inhibitors of host proteases that breach the outer membrane. Here, the X-ray structure of protease-cleaved *Escherichia coli* α-2-macroglobulin is described, which reveals a putative mechanism of activation and conformational change essential for protease inhibition. In this competitive mechanism, protease cleavage of the bait-region domain results in the untethering of an intrinsically disordered region of this domain which disrupts native interdomain interactions that maintain *E. coli* α-2-macroglobulin in the inactivated form. The resulting global conformational change results in entrapment of the protease and activation of the thioester bond that covalently links to the attacking protease. Owing to the similarity in structure and domain architecture of *Escherichia coli* α-2-macroglobulin and human α-2-macro­globulin, this protease-activation mechanism is likely to operate across the diverse members of this group.

## Abstract 2

The X-ray structure of protease-cleaved *E. coli* α-2-macroglobulin is described, which reveals a putative mechanism of activation and conformational change essential for protease inhibition.

## 1. Introduction   

α-2-Macroglobulins (α2Ms) are found in eukaryotic blood, invertebrate haemolymph, the eggs of birds and reptiles, and the bacterial periplasm, where they are thought to play a role in the restriction of proteolytic cleavage (Sottrup-Jensen, 1989 ▸; Lin *et al.*, 2002 ▸; Budd *et al.*, 2004 ▸; Li *et al.*, 2004 ▸; Doan & Gettins, 2008 ▸). Eukaryotic α2Ms have been shown to play important roles in regulating the proteolytic cleavage of a wide range of proteases and are involved in processes such as fibrinolysis and coagulation (De Boer *et al.*, 1993 ▸). Bacterial α2Ms (BA2Ms) are produced by a wide range of Gram-negative bacteria ranging from human pathogenic and commensal strains to plant pathogens and marine bacteria (Budd *et al.*, 2004 ▸). *Escherichia coli* α2M (ECAM) contains the conserved thioester bond that is characteristic of the α2Ms and which is essential for covalent binding to cleaving proteases (Budd *et al.*, 2004 ▸; Doan & Gettins, 2008 ▸; Neves *et al.*, 2012 ▸).

The gene encoding ECAM, *yfhM*, is frequently found in an operon with *pbp1C*, which encodes penicillin-binding protein 1C (Pbp1C; Budd *et al.*, 2004 ▸; Doan & Gettins, 2008 ▸). Pbp1C is predicted to be a bifunctional transpeptidase and transglycosylase owing to its homology to Pbp1A and Pbp1B, which are both essential for the synthesis of the peptidoglycan layer (Schiffer & Höltje, 1999 ▸; Budd *et al.*, 2004 ▸). Both ECAM and Pbp1C are periplasmic proteins that are anchored to the inner membrane and have been proposed to function together in defence and repair against proteases that damage the bacterial cell wall (Budd *et al.*, 2004 ▸). Specifically, it has been postulated that the host proteases produced in defence against bacterial infection, which gain access to the periplasmic space, are inhibited by ECAM, with Pbp1C acting to repair damage (Budd *et al.*, 2004 ▸).

The overall structure of α2Ms comprises a series of β-sheet sandwich macroglobulin (MG) domains forming a ‘keyring’ shape, a bait-region domain (BRD) spanning the body of the ring, a mostly helical thioester domain (TED) connected to the ‘keyring’ by a complement protein subcomponent (CUB) domain, and a C-terminal MG (CTMG) domain (Janssen *et al.*, 2005 ▸; Marrero *et al.*, 2012 ▸; Wong & Dessen, 2014 ▸). Within eukaryotic α2Ms there are eight MG domains including CTMG, which is also known as the receptor-binding domain (MG1–MG7, CTMG), while BA2Ms contain ten MG domains including two N-terminal MG domains anchoring the protein to the inner membrane within the periplasm (MG1–MG9, CTMG) (Huang *et al.*, 1998 ▸; Doan & Gettins, 2007 ▸; Marrero *et al.*, 2012 ▸; Wong & Dessen, 2014 ▸). The TED contains a conserved C*X*EQ motif where a thioester bond is formed, which on activation can covalently link the α2M to lysine residues on the surface of the attacking protease (Sottrup-Jensen *et al.*, 1980 ▸, 1989 ▸; Osterberg & Malmensten, 1984 ▸; Jacobsen & Sottrup-Jensen, 1993 ▸; Janssen *et al.*, 2005 ▸; Abdul Ajees *et al.*, 2006 ▸; Marrero *et al.*, 2012 ▸; Wong & Dessen, 2014 ▸). α2Ms are activated through the protease cleavage of a largely disordered bait region, which results in a conformational change that both traps the protease in a cage-like structure and exposes the highly reactive thioester bond (Travis & Salvesen, 1983 ▸; Sottrup-Jensen, 1989 ▸; Sottrup-Jensen *et al.*, 1989 ▸; Doan & Gettins, 2008 ▸).

In human α2M, chemical cleavage of the thioester bond by methylamine results in a large conformational change that has been interpreted by electron microscopy as similar to that observed on protease cleavage (Sottrup-Jensen *et al.*, 1980 ▸; Tapon-Bretaudiére *et al.*, 1985 ▸; Dodds *et al.*, 1996 ▸; Dodds & Law, 1998 ▸; Marrero *et al.*, 2012 ▸). In contrast, the recently elucidated structures of the BA2M from *Salmonella enterica* serovar Typhimurium (SaA2M) in its unactivated and methylamine-activated forms show that although the overall domain structure of BA2Ms is highly similar to that of human α2M, there is no major conformational change of the bacterial form on chemical cleavage of the thioester bond (Doan & Gettins, 2008 ▸; Neves *et al.*, 2012 ▸; Wong & Dessen, 2014 ▸). In the structures of both bacterial and eukaryotic α2Ms the thioester bond lies close to the surface of the TED, but is protected from hydrolysis by a hydrophobic pocket at the interface between the TED and the CTMG domain (Janssen *et al.*, 2005 ▸; Le *et al.*, 2012 ▸; Wong & Dessen, 2014 ▸).

Owing to a lack of detailed structural information on protease-cleaved forms of α2M, the mechanism through which cleavage of the BRD activates α2M is not known. However, it has been suggested that upon cleavage within human α2M the cleaved BRD interacts with MG2 (MG4 in BA2M), resulting in conformational activation (Marrero *et al.*, 2012 ▸). Alternatively, within human α2M it has been suggested that the bait region interacts with the TED, the MG6 (MG8 in BA2M) and the CUB domains (Marrero *et al.*, 2012 ▸). However, in the absence of a crystal structure of a protease-cleaved form of α2M the mechanism of protease-induced activation remains speculative.

To elucidate the mechanism of protease-induced α2M activation, we crystallized and solved the X-ray structure of a porcine elastase-cleaved form of ECAM, a close homologue of SaA2M (81% amino-acid sequence identity) for which the structure of the unactivated form was recently solved (Wong & Dessen, 2014 ▸). Interestingly, the structure of protease-activated ECAM is highly similar to that of chemically activated human α2M (12% amino-acid sequence identity) and reveals a clear mechanism of how conformational rearrangement is triggered on protease cleavage. Key to activation is the untethering of the intrinsically disordered bait region on cleavage, allowing this disordered region of polypeptide to outcompete the domain–domain interactions that normally maintain the thioester bond in its unactivated form. This suggests a general mechanism through which members of the large and important α2M superfamily are activated.

## 2. Materials and methods   

All chemicals were purchased from Sigma unless mentioned otherwise.

### 2.1. Cloning and protein purification of ECAM   

The gene for ECAM, *yfhM* from *E. coli* K-12, was amplified by PCR and cloned into pET-21a vector using NdeI and XhoI restriction sites. The first 22 residues from the N-terminus of the gene, containing a signal sequence identified using *SignalP*, were excluded from the construct. The stop codon was also excluded, resulting in a protein consisting of residues 23–1631 and a C-terminal 6×His tag (LEHHHHHH). ECAM was initially overexpressed in *E. coli* BL21(DE3) cells and subsequently in T7 Express Crystal Competent *E. coli* cells (methionine-auxotrophic strain, New England Biosciences) using an inducible T7 promoter with 1 m*M* isopropyl β-d-1-thiogalactopyranoside (IPTG) as the inducer. Bacteria expressing ECAM were grown in lysogeny broth; selenomethionine-labelled ECAM was obtained using M9 minimal medium supplemented with 50 mg l^−1^ selenomethionine and 20 mg l^−1^ of each of nine essential amino acids (excluding methionine). Cells were grown at 37°C to an OD_600_ of 0.6, protein production was induced by the addition of 1 m*M* IPTG and the cells were grown for a further 6 h. The cell pellet was collected by centrifugation at 4400*g* for 15 min and the cells were resuspended in binding buffer [20 m*M* Tris, 10 m*M* imidazole, 500 m*M* sodium chloride, 5 m*M* tris(2-carboxy­­ethyl)phosphine (TCEP) pH 7.5] and lysed by sonication with 1 mg ml^−1^ lysozyme in the presence of protease inhibitors (Complete Mini, Roche). Cell debris was removed by centrifugation at 46 000*g* for 30 min at 4°C. The cell supernatant was then loaded onto a HisTrap HP column (GE Healthcare) and the bound protein was eluted with elution buffer (20 m*M* Tris, 500 m*M* imidazole, 500 m*M* sodium chloride, 5 m*M* TCEP pH 7.5) using a linear gradient increasing from 10 to 500 m*M*. Fractions containing ECAM were pooled and dialysed overnight at 4°C into 50 m*M* Tris, 200 m*M* sodium chloride pH 7.5 and run on a Superdex S200 gel-filtration column (GE Healthcare). Central fractions from the peak were combined and concentrated using a 100 kDa molecular-weight cutoff centrifugal concentrator.

### 2.2. Crystallization and structure building   

Purified ECAM was reacted in a 1:1 molar ratio with porcine elastase (MP Biomedicals) in 50 m*M* Tris, 200 m*M* NaCl pH 7.5 on ice for 5 min before being loaded onto a Superdex S200 gel-filtration column (GE Healthcare). The two major peaks from gel filtration were concentrated to 16 mg ml^−1^ separately using 100 kDa molecular-weight cutoff centrifugal concentrators and used in crystallization trials. Several hundred crystallization conditions were tested, including the JCSG-plus, MIDAS and Morpheus screens (Molecular Dimensions), for both concentrated peaks. A Cartesian Honeybee 8+1 (Harvard Bioscience) robot was used with 96-well plates, dispensing 0.5 µl reservoir solution and 0.5 µl protein sample. Subsequent scaled-up crystal growth was performed using 2.5 µl reservoir solution and 2.5 µl protein sample. The initial crystal was grown in conditions consisting of 0.1 *M* potassium chloride, 0.1 *M* HEPES, 25% Sokalan CP 7 pH 7.0, and upon optimization the pH was adjusted to 7.5 for larger crystal growth. Crystals were grown using equal volumes of protease-cleaved ECAM and reservoir solution using sitting-drop vapour diffusion, with crystals appearing after 2 d at 16°C for the second fraction and after two weeks for the first fraction. Cryoprotection was optimized with a 3:2 ratio of xylitol-saturated reservoir solution to reservoir solution. Crystals were briefly soaked and flash-cooled in liquid nitrogen for data collection. The best diffraction resolution obtained was 3.8 Å, and molecular replacement with methylamine-activated α2M (PDB entry 4acq) was unsuccessful, most likely as the sequence identity with the human homologue was low (12%) and owing to the difference in domain orientation between the structural models (Marrero *et al.*, 2012 ▸). Further expression was performed using a methionine-auxotrophic strain of *E. coli* BL21 (T7 Express Crystal Competent *E. coli*, New England Bioscience) and the purification and crystallization screens were repeated using selenomethionine-labelled protein. As repeating the previous protocol with selenomethionine-labelled protein was unsuccessful, *in situ* proteolytic cleavage screening was performed using porcine elastase. Successful crystallization was achieved using a 1:100 ratio of porcine elastase to selenomethionine-labelled ECAM. Crystallization was successful in the same condition as used previously, with the crystal having a similar appearance and the same space group as previous unlabelled crystals. These crystals diffracted to 3.65 Å resolution and phases were obtained using single-wavelength anomalous diffraction (SAD).

Data were collected for ECAM crystals on the I02, I03 and I24 beamlines at Diamond Light Source, Didcot, England at 100 K at the Se *K* edge (λ = 0.97939 Å) using a PILATUS 6M detector. A high-redundancy SAD data set was processed and scaled using *XDS* and *AIMLESS* from the *CCP*4 suite of programs (Evans, 2006 ▸; Kabsch, 2010 ▸; Winn *et al.*, 2011 ▸). Selenium sites were located using *SHELXC*/*D*, with the best substructure solution consisting of 23 sites (Sheldrick, 2010 ▸). These selenium sites were input along with the SAD data set to *AutoSol* within the *PHENIX* package to perform phasing and density modification (Terwilliger *et al.*, 2009 ▸). Density modification in *AutoSol* was sufficient to break the phase ambiguity owing to the high solvent content of the crystal (69%). This yielded interpretable, low-resolution maps in which density corresponding to secondary-structure elements and larger amino-acid side chains was visible. Initially, the α-helical TED domain was built in *Coot* using idealized α-helical sections (Emsley *et al.*, 2010 ▸). The loops between these sections were connected where density was available. The six selenomethionine sites in this domain provided the starting sites for the building of amino-acid side chains in the TED domain, although owing to the resolution initially only larger side chains and those where continuous sequence could be determined were built. In addition to the building of the TED domain, a number of α2M-derived polyalanine MG domains were rigid-body fitted into their corresponding density and manually real-space refined in *Coot* (Emsley *et al.*, 2010 ▸). As with the TED domain, where possible side chains were fitted using the positions of selenomethionine Se atoms as starting sites. This initial building yielded a partial model, which was then used in conjunction with the selenomethionine substructure to rephase the experimental data using MR-SAD phasing in *Phaser* (McCoy *et al.*, 2007 ▸). This process led to phase improvement and the appearance of new features in the map, which were modelled, and the process was repeated iteratively. Partway through the building process the atomic coordinates for SaA2M were published (PDB entry 4u48; Wong & Dessen, 2014 ▸), and the domains from this model provided validation of the MG-domain placement and side-chain modelling in our experimentally phased map. The SaA2M structure also provided a template for building the more difficult sections of the model. At this point restrained TLS refinement using *REFMAC*5 was found to stably improve both *R*_work_ and *R*_free_, and refinement was performed and the model was improved and finished manually in *Coot* (Murshudov *et al.*, 2011 ▸; Emsley *et al.*, 2010 ▸). Electron density for the thioester bond indicated that the deaminated glutamine forms no covalent bond to the cysteine. Before submission of the final model, the quality of the structure was assessed using the *MolProbity* webserver (Chen *et al.*, 2010 ▸). The atomic coordinates and structure factors were deposited in the Protein Data Bank (PDB entry 4rtd). Statistics for data collection, experimental phasing and refinement are presented in Table 1 ▸. For mass-spectrometric analysis of protease-cleaved ECAM, crystals were washed in reservoir solution before being dissolved in deionized water and heated to 96°C in bromophenol blue sample buffer for 5 min. The sample was then run on a NuPAGE Novex 4–12% bis-tris gel (Invitrogen) and visible bands were cut for proteomic analysis. Samples were digested by trypsin and analysed by LC-MS/MS (Orbitrap XL) performed at the Fingerprints Proteomics Facility at the University of Dundee.

## 3. Results   

### 3.1. Overall structure of protease-activated ECAM   

To determine the structural changes that occur on protease cleavage of ECAM, we performed protease digestion with porcine elastase and used the major products from gel filtration of cleaved ECAM to perform crystallization trials. This yielded diffracting crystals in 0.1 *M* potassium chloride, 0.1 *M* HEPES, 25% Sokalan CP 7 pH 7.0 with cleaved ECAM. In order to obtain phase information, we attempted to repeat this process with selenomethionine-labelled ECAM, but this failed to yield crystals. However, an alternative strategy of *in situ* proteolysis and crystallization with selenomethionine-labelled ECAM was successful. This method yielded crystals that diffracted to 3.65 Å resolution. Upon completion and validation of the model of protease-activated ECAM, the number of Ramachandran outliers remaining was appropriate for a crystal structure with a resolution of 3.65 Å. Although the *R*_merge_ and *R*_p.i.m._ values were high, this can be explained by the highly redundant data set used; with a CC_1/2_ of 0.675 in the highest shell, these data used were judged to be acceptable (Karplus & Diederichs, 2012 ▸; Diederichs & Karplus, 2013 ▸).

**Figure 1:**
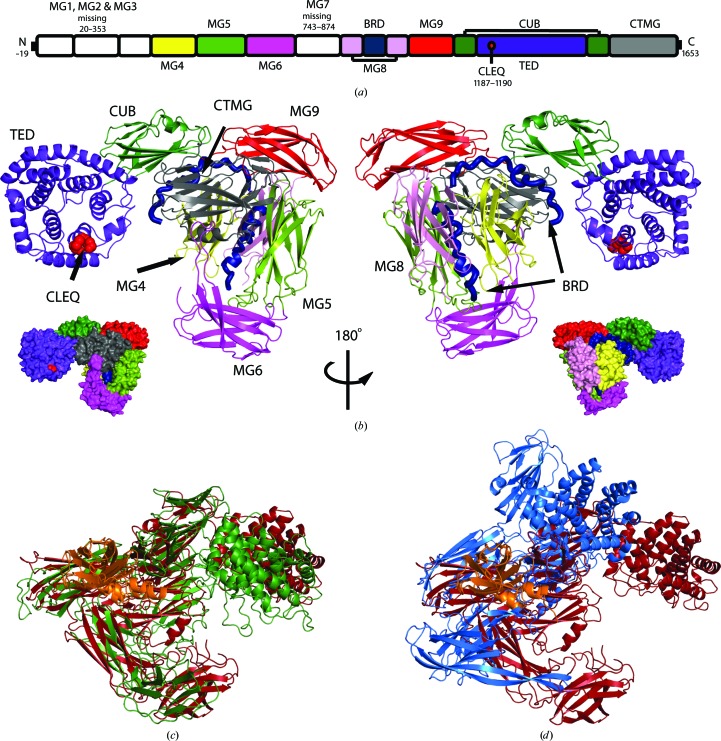
Crystal structure of porcine elastase-cleaved *E. coli* α2M (ECAM). (*a*) Schematic representation of the 13 domains of ECAM showing the macroglobulin domains (MG) including the C-terminal MG (CTMG) domain, the bait-region domain (BRD), the complement protein subcomponent (CUB) domain and the thioester domain (TED) containing the CLEQ motif. (*b*) The structure of elastase-cleaved ECAM with the individual domains coloured as in (*a*). Smaller van der Waals surfaces in both views are also presented. In the left view the α-helical TED is orientated with the CLEQ thioester (drawn as red van der Waals spheres) positioned above the pocket which is thought to accommodate the attacking protease. (*c*) Structural alignment of methylamine-treated human α2M (PDB entry 4acq monomer trimmed to the domains present in cleaved ECAM) and elastase-cleaved ECAM (PDB entry 4rtd) in green and red, respectively. (*d*) Structural alignment of native SaA2M (PDB entry 4u48 trimmed to the domains present in cleaved ECAM) and elastase-cleaved ECAM (PDB entry 4rtd) in blue and red, respectively. Structural alignments were performed using the MG domain containing the bait region (shown in orange).

Similar to the domain architecture of native SaA2M, uncleaved ECAM consists of ten MG domains, with a largely disordered bait-region domain found within MG8, a TED that houses the reactive thioester bond and a CUB domain (Fig. 1 ▸ *a*). Elastase-cleaved ECAM (Fig. 1 ▸ *b*) adopts a conformation similar to that of methylamine-activated human α2M (Fig. 1 ▸ *c*) but distinct from the unactivated form of SaA2M (Fig. 1 ▸ *d*). Despite the low sequence identity between human α2M and ECAM (12%), the r.m.s.d. between C^α^ atoms for these proteins is 14.1 Å, while that for SaA2M and ECAM, which share 82% sequence identity, is 22.1 Å. Most notably, in the structure of the protease-cleaved ECAM, interactions between the TED and the CTMG domain, which protects the thioester bond in unactivated SaA2M, are not present. Instead, the thioester region of TED is solvent-exposed and faces the expected location of the attacking protease, as it would be positioned when cleaving the bait region of ECAM (Fig. 1 ▸ *b*). Electron density for elastase, in addition to that for MG domains 1, 2, 3 and 7, was absent in 2*F*_o_ − *F*_c_ maps of cleaved ECAM, as was electron density for 20 amino-acid residues (Arg923–Leu942) within the bait region. Analysis of elastase-cleaved ECAM by SDS–PAGE and mass spectrometry confirms that MG domains 1, 2, 3 and 7 are absent from the crystallized protein (Supplementary Fig. S1). Two bands at 90 and 75 kDa on SDS–PAGE have similar peptide coverage, encompassing the same domains (Supplementary Fig. S1). However, the reason for the difference in apparent molecular weights between these two species is not known.

### 3.2. Interaction of the BRD with the CTMG domain   

**Figure 2:**
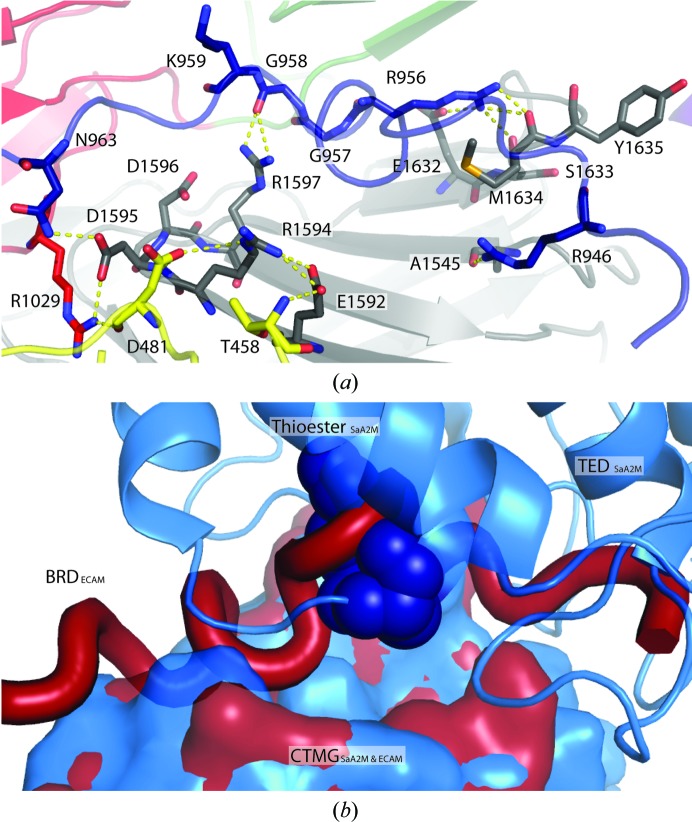
Bait-region interaction with the CTMG domain. (*a*) Here we show hydrogen bonding between the cleaved bait region (blue) and the CTMG domain (grey) as dashed yellow lines. Note the interaction between Arg956 and Met1634, with the CTMG methionine normally involved in the hydrophobic pocket found in uncleaved native SaA2M. MG4 and MG9 are shown in yellow and red, respectively. (*b*) Surface representation of the CTMG domain bound to the TED in SaA2M (blue) overlaid (by superposition of CTMG domains) with the previously disordered bait region bound to the CTMG domain in elastase-cleaved ECAM (red). The thioester bond within the TED is shown as dark blue van der Waals spheres.

**Figure 3:**
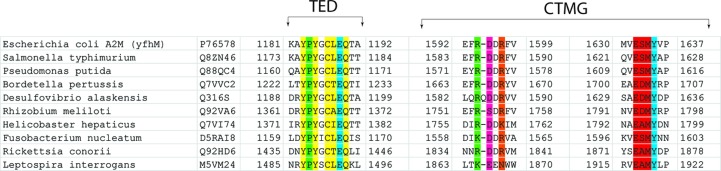
Sequence alignments of conserved motifs involved in the thioester pocket and conformational activation. Highlighted in yellow are tyrosines important for maintaining the hydrophobic pocket protecting the thioester bond (CLEQ motif, also shown in yellow). The tyrosine within the C-terminal macroglobulin (CTMG) domain, involved in protecting the thioester, which coordinates to the glutamate within the CLEQ motif in the native SaA2M structure is highlighted in blue (PDB entry 4u48). The conserved proline found near the thioester is highlighted in green along with the arginine that it coordinates in unactivated SaA2M. The residues highlighted in pink, orange and red in the CTMG domain coordinate to residues Asn963, Gly958 and Arg956, respectively, in the cleaved bait region of elastase-cleaved ECAM.

Although it is known that protease cleavage of the largely disordered bait region of both human and bacterial α2Ms gives rise to a large conformational rearrangement and activation of the thioester bond, the mechanism through which this is mediated was unclear (Barrett *et al.*, 1979 ▸; Neves *et al.*, 2012 ▸). However, the structure of protease-cleaved ECAM provides a clear mechanism for this process. Thioester bond release is achieved by the untethering of a large region of an unstructured polypeptide chain upon cleavage of the BRD that forms new interactions with the CTMG domain, thereby preventing the protective interaction with the TED. Specifically, in the elastase-cleaved form of ECAM additional residues (Phe947–Asn963) of the BRD are observed that are disordered in the uncleaved SaA2M structure. All of these residues in the protease-cleaved ECAM structure are ordered owing to the formation of a new binding interface between the BRD and the CTMG domain upon protease cleavage (Fig. 2 ▸ *a*). Critically, the region of the CTMG domain that is involved in BRD binding substantially overlaps with the region of the CTMG domain that forms the binding interface with the TED in the uncleaved form of the protein (Fig. 2 ▸ *b*). The buried surface area between the CTMG domain and TED in SaA2M is 1972 Å^2^, with three hydrogen bonds between these domains, whereas the buried surface area between the elastase-cleaved BRD and the CTMG domain in ECAM is 1438 Å^2^, with five hydrogen bonds between the domains. In the protease-cleaved ECAM, hydrogen-bond formation between residues of the CTMG domain and the cleaved bait region involves the highly conserved RDDR and E*X*MY motifs (Fig. 3 ▸). Interestingly, it is residues within these motifs that also form hydrogen bonds to the TED in the uncleaved SaA2M protein (Fig. 3 ▸; Wong & Dessen, 2014 ▸).

### 3.3. Cleavage-induced conformational changes and thioester-bond activation of α2Ms   

**Figure 4:**
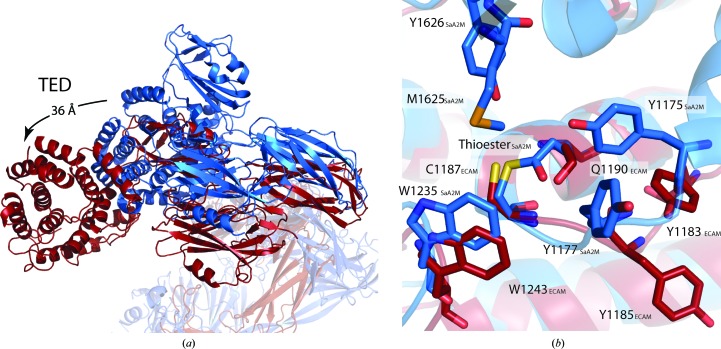
Conformational shift of the TED and thioester of protease-activated ECAM. (*a*) Superposition of protease-cleaved ECAM (red) and SaA2M (blue) showing a 36 Å shift of the TED. Structures were overlaid by superposition of MG8 containing the BRD. (*b*) Overlaid elastase-cleaved ECAM (red) and native SaA2M (blue) TEDs. Tyrosines thought to protect the thioester region from hydrolysis in native SaA2M (Tyr1175 and Tyr1177) are orientated away from the protease-activated ECAM thioester region (Tyr1183 and Tyr1185 in protease-activated ECAM).

We propose that the loss of the interaction of the TED with the CTMG domain is sufficient to enable both the large TED movement observed in the cleaved ECAM structure relative to the unactivated SaA2M structure and the exposure of the thioester bond (Fig. 4 ▸ *a*, Supplementary Movie S1). The movement of the TED domain shows an overall shift of 36 Å, with the MG6 domain moving by 50 Å and with both the MG6 and TED arms hugging the position at which the attacking protease would be located to cleave the BRD (Fig. 4 ▸ *a*, Supplementary Movie S1). In addition to these global conformational changes, protease cleavage leads to localized changes in the environment of the thioester bond that are likely to lead to its activation. In the unactivated SaA2M structure the conserved methionine and tyrosine side chains (Met1625 and Tyr1626 in SaA2M) from the CTMG domain form part of the hydrophobic pocket at the interface with the TED that has been shown to be important in maintenance of the thioester bond (Wong & Dessen, 2014 ▸). The loss of these key side-chain interactions and the movement of the additional side chains which constitute the protective hydrophobic pocket (Tyr1175 and Tyr1177 in SaA2M and Tyr1183 and Tyr1185 in ECAM) from the TED exposes the thioester bond to the solvent, allowing hydrolysis or covalent-bond formation with the attacking protease (Fig. 4 ▸ *b*). As there are no contacts present from the CTMG domain providing a hydrophobic pocket to protect the thioester from hydrolysis by water, the thioester bond could be cleaved and the deaminated glutamine (Gln1190) would be converted to a glutamic acid. Within our protease-activated ECAM structure the distance between the S atom of Cys1187 and the C^γ^ atom of Gln1190 is 4.6 Å, indicating that the thioester bond may not be intact (Supplementary Fig. S2). However, we cannot rule out the possibility that there is a mixed population of molecules, some of which possess an intact thioester bond. Owing to this ambiguity, we have represented the thioester without a covalent bond between Cys1187 and the C^γ^ atom of Gln1190 and have also omitted the O atom from the deaminated glutamine that would be formed on hydrolysis of the thioester bond.

## 4. Discussion   

Although it has been suggested that small-molecule-activated human α2M resembles the conformation of the protease-cleaved form, there have to date been no detailed structural data to confirm this (Tapon-Bretaudiére *et al.*, 1985 ▸). The similarity in the overall conformation of protease-cleaved ECAM to that of methylamine-activated human α2M confirms that the protease-activated and chemically activated forms of human α2M are structurally equivalent. In addition, these data suggest that the entrapment of cleaving proteases is likely to occur in a similar manner for BA2Ms as has been proposed for human α2M (Marrero *et al.*, 2012 ▸; Meyer *et al.*, 2012 ▸). However, there are clearly key differences in the details of the interactions that maintain the inactive conformations of BA2Ms and human α2M, since the recent structures of unactivated and methylamine-activated SaA2M show that chemical cleavage of the thioester bond does not lead to global conformational changes in this case (Marrero *et al.*, 2012 ▸; Wong & Dessen, 2014 ▸). This difference is likely to be owing to the domain location of the side chains that comprise the thioester-protecting pocket (Supplementary Fig. S3). In BA2Ms this pocket comprises two tyrosine side chains from the TED and a tyrosine and a methionine side chain from the CTMG domain, whereas in eukaryotic α2Ms all four residues (three tyrosine side chains and one methionine side chain) are found in the CTMG domain. When SaA2M is reacted with methyl­amine no conformational change is seen, but Tyr1175 from the TED is displaced (Wong & Dessen, 2014 ▸). The structural counterpart of this side chain (Tyr1307 from TEP1) in eukaryotic α2M family members is, however, found within the CTMG domain (Supplementary Fig. S3; Baxter *et al.*, 2007 ▸). It is presumably the rearrangement of this side chain and perhaps other CTMG side chains that comprise the thioester-protecting pocket which leads to the loss of TED–CTMG domain binding and subsequent global conformational changes. The buried surface area seen between the TED and the CTMG domain in native SaA2M decreases by 27% compared with that of the BRD and the CTMG domain in protease-cleaved ECAM; however, the number of hydrogen bonds increases. Although there is a decrease in the buried surface area, it is the interaction between important residues for protecting the thioester that would be expected to trigger conformational change releasing TED and allow subsequent interaction with a cleaving protease.

**Figure 5:**
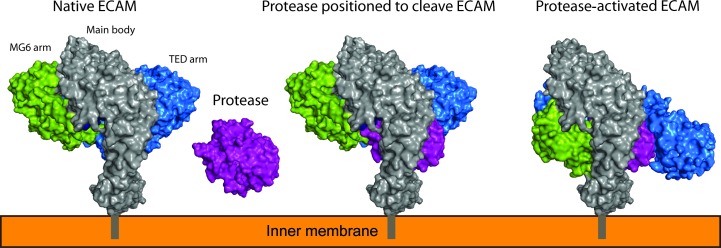
Putative mechanism of protease entrapment and inhibition by ECAM. ECAM with a membrane anchor within the periplasm of bacteria encounters protease and forms a covalent complex upon cleavage, inhibiting the protease from the cleavage of large substrates. The main body of ECAM anchored to the inner membrane is shown in grey and contains the domains showing little movement (MG4, MG8 and BRD) between native and protease-activated structures or that are not present in the protease-activated structure (MG1–MG3 and MG7). The blue TED arm (TED, CUB, CTMG and MG9 domains) and green MG6 arm (MG5 and MG6 domains) entrap the protease with the blue arm containing the thioester that forms a covalent bond.

The role of BA2Ms, which are inner membrane-anchored periplasmic proteins, has been suggested as protease inhibitors that inhibit exogenous proteases that have breached the outer membrane. Comparison of the structures of unactivated SaA2M and protease-cleaved ECAM illustrates how protease-induced conformational changes enable protease entrapment (Fig. 5 ▸). The movement of the TED-domain arm and MG6-domain arm around the central pocket above the bait region is similar to the movement seen in methylamine-activated human α2M; when accounting for the domains that are not present in the protease-activated ECAM structure no clashes are seen between the moving arms and the absent domains (Marrero *et al.*, 2012 ▸). The entrapment of proteases would limit the proteolysis to smaller substrates, as has been suggested for human α2M, and would prevent the cleavage of important larger substrates such as the peptide component of the peptidoglycan layer or large proteins (Fig. 5 ▸; Sottrup-Jensen, 1989 ▸). Although we cannot be sure why the covalently bound elastase is not present in our structure, this may be owing to a lack of available and correctly positioned lysine side chains on the surface of the protease, since the thioester bond is preferentially cleaved by this side chain (Marrero *et al.*, 2012 ▸). The lack of MG domains 1, 2, 3 and 7 within the crystal lattice is likely to be owing to the MG domains being cleaved by the elastase.

In summary, the structure of protease-activated ECAM shows a competitive mechanism of activation in which cleavage of the BRD allows the normally intrinsically disordered region of this domain to outcompete the TED for CTMG-domain binding. Loss of the TED–CTMG domain interaction leads to a large conformational rearrangement of ECAM and exposure of the reactive thioester bond. The structural similarity between methylamine-activated human α2M and protease-cleaved ECAM suggests that similar mechanisms are likely to operate across the diverse members of the α2M family.

## Supplementary Material

PDB reference: *Escherichia coli* α-2-macroglobulin activated by porcine elastase, 4rtd

Supplementary Figures S1-S3.. DOI: 10.1107/S1399004715008548/mn5091sup1.pdf

Supplementary Movie S1. Animation of the domain rearrangement upon cleavage of ECAM. The animation shows the movement of domains in ECAM when cleaved by a protease. Upon cleavage of the BRD, the TED arm and MG8 arm move around the central pocket, becoming the final protease-activated form (PDB entry 4rtd combined with missing domains from PDB entry 4u48, superpositioned using the MG8 domain).. DOI: 10.1107/S1399004715008548/mn5091sup5.mp4

## Figures and Tables

**Table 1:** Data-collection and refinement statistics (single-wavelength anomalous diffraction) for protease-cleaved ECAM Data were collected from one crystal. Values in parentheses are for the highest resolution shell.

Data collection	
Space group	*H*3
Unit-cell parameters (, )	*a* = *b* = 176.06, *c* = 161.13, = = 90, = 120
Resolution ()	46.873.65 (4.003.65)
Solvent content (%)	69
No. of reflections	20753 (4991)
CC_1/2_	0.998 (0.675)
*R*_merge_ (%)	39.0 (378.0)
*R*_p.i.m._ † (%)	7.7 (74.4)
*I*/(*I*)	13.2 (2.1)
Completeness (%)	99.9 (99.9)
Multiplicity	26.6 (26.7)
Anomalous completeness (%)	99.9 (99.3)
Anomalous multiplicity	12.8 (12.8)
DelAnom correlation between half sets	0.335 (0.011)
Mid-slope of anomalous normal probability	1.245
Refinement	
*R*_work_/*R*_free_ (%)	17.7/23.8
No. of atoms	8699
Average *B* factor (^2^)	144
R.m.s. deviations	
Bond lengths ()	0.01
Bond angles ()	1.53
Ramachandran plot‡ (%)	
Favoured	90.7
Allowed	7.9
Outliers	1.3
PDB code	4rtd

† *R*_p.i.m._ = 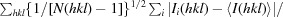 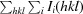.

‡ Percentages of residues in favoured/allowed regions calculated by *MolProbity* (Chen *et al.*, 2010 ▸).

## References

[bb1] Abdul Ajees, A., Gunasekaran, K., Volanakis, J. E., Narayana, S. V. L., Kotwal, G. J. & Krishna Murthy, H. M. (2006). *Nature (London)*, **444**, 221–225. 10.1038/nature05258 17051152

[bb2] Barrett, A. J., Brown, M. A. & Sayers, C. A. (1979). *Biochem. J.* **181**, 401–418. 10.1042/bj1810401 PMC1161172 91367

[bb3] Baxter, R. H. G., Chang, C.-I., Chelliah, Y., Blandin, S., Levashina, E. A. & Deisenhofer, J. (2007). *Proc. Natl Acad. Sci. USA*, **104**, 11615–11620. 10.1073/pnas.0704967104 PMC1905922 17606907

[bb4] Boer, J. P. de, Creasey, A. A., Chang, A., Abbink, J. J., Roem, D., Eerenberg, A. J., Hack, C. E. & Taylor, F. B. (1993). *Infect. Immun.* **61**, 5035–5043. 10.1128/iai.61.12.5035-5043.1993 PMC281280 7693593

[bb5] Budd, A., Blandin, S., Levashina, E. A. & Gibson, T. J. (2004). *Genome Biol.* **5**, R38. 10.1186/gb-2004-5-6-r38 PMC463071 15186489

[bb6] Chen, V. B., Arendall, W. B., Headd, J. J., Keedy, D. A., Immormino, R. M., Kapral, G. J., Murray, L. W., Richardson, J. S. & Richardson, D. C. (2010). *Acta Cryst.* D**66**, 12–21. 10.1107/S0907444909042073 PMC2803126 20057044

[bb7] Diederichs, K. & Karplus, P. A. (2013). *Acta Cryst.* D**69**, 1215–1222. 10.1107/S0907444913001121 PMC3689524 23793147

[bb8] Doan, N. & Gettins, P. G. W. (2007). *Biochem. J.* **407**, 23–30. 10.1042/BJ20070764 PMC2267405 17608619

[bb9] Doan, N. & Gettins, P. G. W. (2008). *J. Biol. Chem.* **283**, 28747–28756. 10.1074/jbc.M803127200 PMC2568910 18697741

[bb10] Dodds, A. W. & Law, S. K. A. (1998). *Immunol. Rev.* **166**, 15–26. 10.1111/j.1600-065x.1998.tb01249.x 9914899

[bb11] Dodds, A. W., Ren, X.-D., Willis, A. C. & Law, S. K. A. (1996). *Nature (London)*, **379**, 177–179. 10.1038/379177a0 8538770

[bb12] Emsley, P., Lohkamp, B., Scott, W. G. & Cowtan, K. (2010). *Acta Cryst.* D**66**, 486–501. 10.1107/S0907444910007493 PMC2852313 20383002

[bb13] Evans, P. (2006). *Acta Cryst.* D**62**, 72–82. 10.1107/S0907444905036693 16369096

[bb14] Huang, W., Dolmer, K., Liao, X. & Gettins, P. G. W. (1998). *Protein Sci.* **7**, 2602–2612. 10.1002/pro.5560071214 PMC2143881 9865955

[bb15] Jacobsen, L. & Sottrup-Jensen, L. (1993). *Biochemistry*, **32**, 120–126. 10.1021/bi00052a017 7678194

[bb16] Janssen, B. J. C., Huizinga, E. G., Raaijmakers, H. C. A., Roos, A., Daha, M. R., Nilsson-Ekdahl, K., Nilsson, B. & Gros, P. (2005). *Nature (London)*, **437**, 505–511. 10.1038/nature04005 16177781

[bb17] Kabsch, W. (2010). *Acta Cryst.* D**66**, 125–132. 10.1107/S0907444909047337 PMC2815665 20124692

[bb18] Karplus, P. A. & Diederichs, K. (2012). *Science*, **336**, 1030–1033. 10.1126/science.1218231 PMC3457925 22628654

[bb19] Le, B. V., Williams, M., Logarajah, S. & Baxter, R. H. G. (2012). *PLoS Pathog.* **8**, e1002958. 10.1371/journal.ppat.1002958 PMC3464232 23055931

[bb20] Li, Z.-F., Wu, X.-H. & Engvall, E. (2004). *Genomics*, **83**, 1083–1093. 10.1016/j.ygeno.2003.12.005 15177561

[bb21] Lin, M., Sutherland, D. R., Horsfall, W., Totty, N., Yeo, E., Nayar, R., Wu, X.-F. & Schuh, A. C. (2002). *Blood*, **99**, 1683–1691. 10.1182/blood.v99.5.1683 11861284

[bb22] Marrero, A., Duquerroy, S., Trapani, S., Goulas, T., Guevara, T., Andersen, G. R., Navaza, J., Sottrup-Jensen, L. & Gomis-Rüth, F. X. (2012). *Angew. Chem. Int. Ed.* **51**, 3340–3344. 10.1002/anie.201108015 22290936

[bb23] McCoy, A. J., Grosse-Kunstleve, R. W., Adams, P. D., Winn, M. D., Storoni, L. C. & Read, R. J. (2007). *J. Appl. Cryst.* **40**, 658–674. 10.1107/S0021889807021206 PMC2483472 19461840

[bb24] Meyer, C., Hinrichs, W. & Hahn, U. (2012). *Angew. Chem. Int. Ed.* **51**, 5045–5047. 10.1002/anie.201201104 22488953

[bb35] Murshudov, G. N., Skubák, P., Lebedev, A. A., Pannu, N. S., Steiner, R. A., Nicholls, R. A., Winn, M. D., Long, F. & Vagin, A. A. (2011). *Acta Cryst.* D**67**, 355–367. 10.1107/S0907444911001314 PMC3069751 21460454

[bb25] Neves, D., Estrozi, L. F., Job, V., Gabel, F., Schoehn, G. & Dessen, A. (2012). *PLoS One*, **7**, e35384. 10.1371/journal.pone.0035384 PMC3328433 22530012

[bb26] Osterberg, R. & Malmensten, B. (1984). *Eur. J. Biochem.* **143**, 541–544. 10.1111/j.1432-1033.1984.tb08403.x 6207020

[bb28] Schiffer, G. & Höltje, J. V. (1999). *J. Biol. Chem.* **274**, 32031–32039. 10.1074/jbc.274.45.32031 10542235

[bb29] Sheldrick, G. M. (2010). *Acta Cryst.* D**66**, 479–485. 10.1107/S0907444909038360 PMC2852312 20383001

[bb30] Sottrup-Jensen, L. (1989). *J. Biol. Chem.* **264**, 11539–11542. 2473064

[bb31] Sottrup-Jensen, L., Petersen, T. E. & Magnusson, S. (1980). *FEBS Lett.* **121**, 275–279. 10.1016/0014-5793(80)80361-9 6161841

[bb32] Sottrup-Jensen, L., Sand, O., Kristensen, L. & Fey, G. H. (1989). *J. Biol. Chem.* **264**, 15781–15789. 2476433

[bb33] Tapon-Bretaudiére, J., Bros, A., Couture-Tosi, E. & Delain, E. (1985). *EMBO J.* **4**, 85–89. 10.1002/j.1460-2075.1985.tb02321.x PMC554155 16453601

[bb34] Terwilliger, T. C., Adams, P. D., Read, R. J., McCoy, A. J., Moriarty, N. W., Grosse-Kunstleve, R. W., Afonine, P. V., Zwart, P. H. & Hung, L.-W. (2009). *Acta Cryst.* D**65**, 582–601. 10.1107/S0907444909012098 PMC2685735 19465773

[bb27] Travis, J. & Salvesen, G. S. (1983). *Annu. Rev. Biochem.* **52**, 655–709. 10.1146/annurev.bi.52.070183.003255 6193754

[bb36] Winn, M. D. *et al.* (2011). *Acta Cryst.* D**67**, 235–242.

[bb37] Wong, S. G. & Dessen, A. (2014). *Nature Commun.* **5**, 4917. 10.1038/ncomms5917 25221932

