# Supplementary material for: Structure of protease-cleaved Escherichia coli α-2-macroglobulin reveals a putative mechanism of conformational activation for protease entrapment
Source: Acta Crystallogr D Biol Crystallogr. 2015 Jun 30;71(Pt 7):1478–86. doi: 10.1107/S1399004715008548 (PMC4498604; doi:10.1107/S1399004715008548)
Supplement: Supplementary file 1 [file d-71-01478-sup1.pdf]

BIOLOGICAL
CRYSTALLOGRAPHY

Volume 71 (2015)

Supporting information for article:

Structure of protease-cleaved *Escherichia coli* α-2-macroglobulin reveals a putative mechanism of conformational activation for protease entrapment

Cameron D. Fyfe, Rhys Grinter, Inokentijs Josts, Khedidja Mosbahi, Aleksander w. Roszak, Richard J. Cogdell, Daniel M. Wall, Richard J. S. Burchmore, Olwyn Byron and Daniel Walker

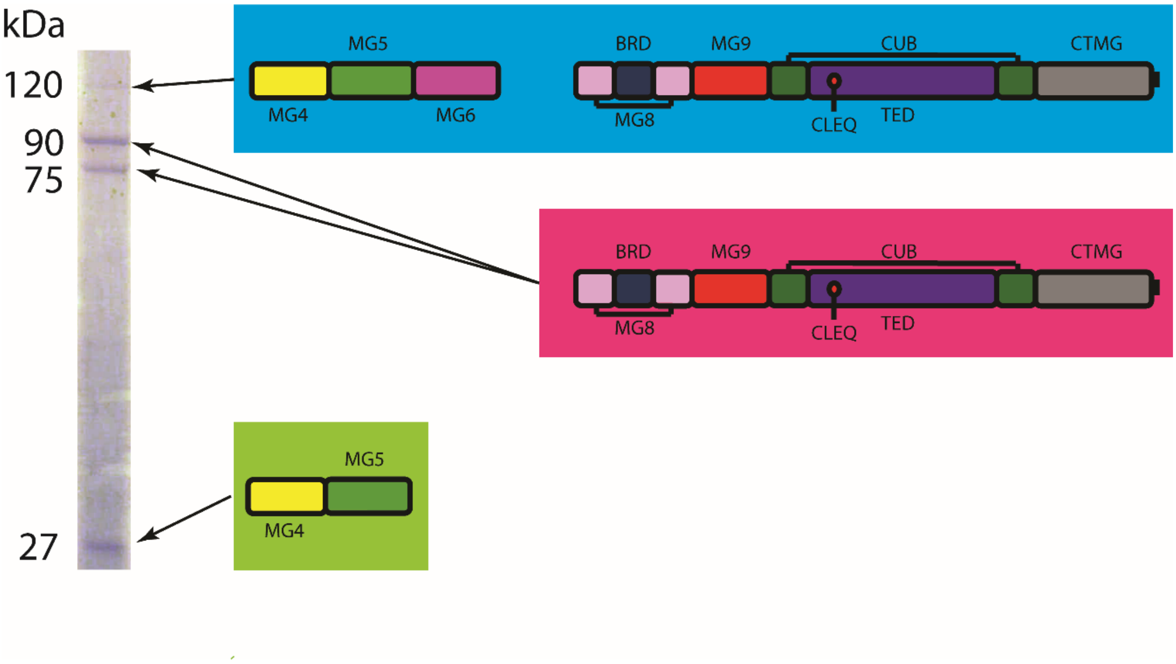

Figure S1 Elastase treated ECAM crystals on SDS PAGE and LC MSMS peptide coverage from bands. Peptide coverage was found from the corresponding domains upon trypsin digestion and LC MSMS. The band at 120 kDa (blue) had peptide coverage from the all domains seen in the crystal structure and the bands at 90 and 75 kDa (pink) had peptide coverage from the N terminus seen in the crystal structure. The band seen at 27 kDa (green) had peptide coverage from domains MG4 and 5.

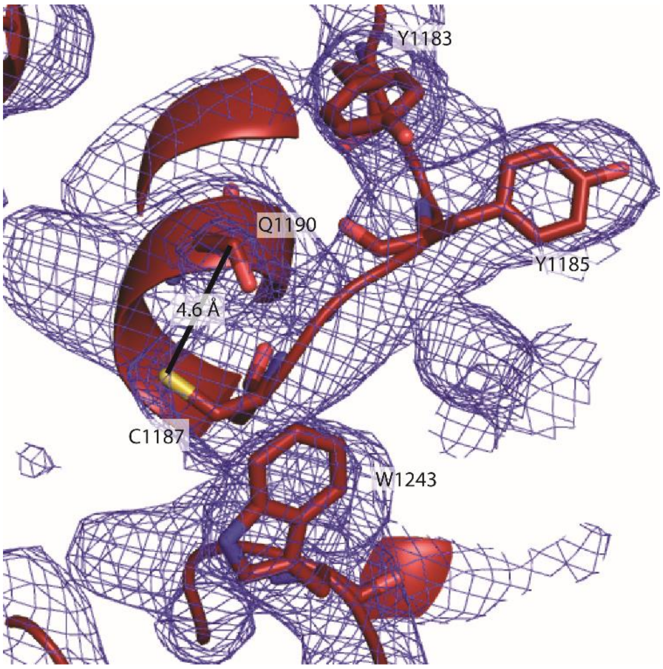

Figure S2 Cleaved thioester of protease-cleaved ECAM. The 2 Fo-Fc electron density contoured at 1 σ level for protease cleaved ECAM. The S-Cγ distance between C1187 and Q1190 is seen to be 4.6 Å, suggestive of a cleaved thioester bond.

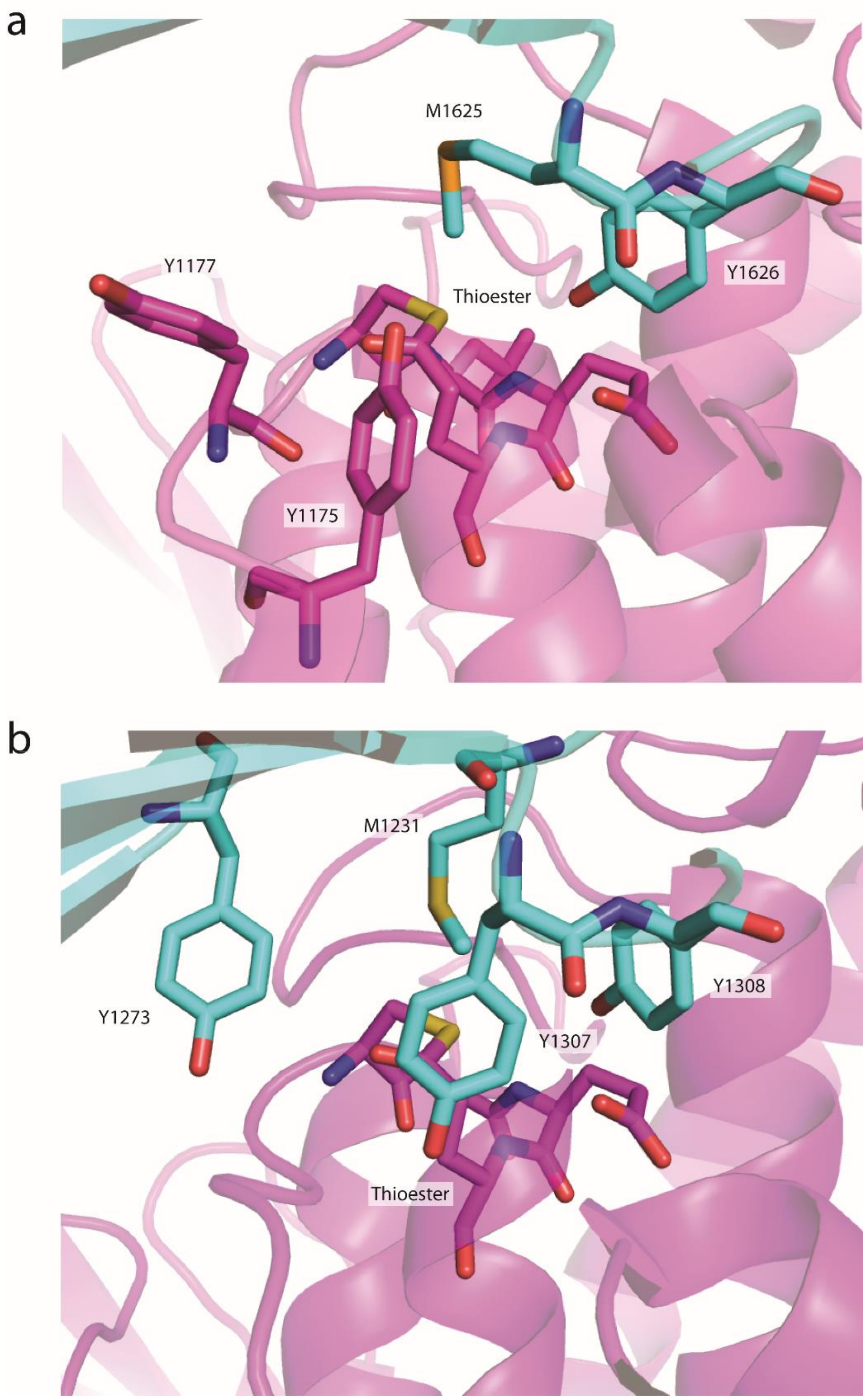

Figure S3 Comparison of the thioester protecting pocket in eukaryotic and bacterial α2Ms. The thioester pocket highlighting conserved residues from the TED (magenta) and CTMG domain (cyan) of (a) SaA2M and (b) the eukaryotic α2M family member TEP1 (PDB 4D94; Le *et al.*, 2012). Highlighted side chains are conserved among bacterial and eukaryotic α2Ms, respectively.
